# Crosslinked duplex DNA nanogels that target specified proteins

**DOI:** 10.1080/14686996.2016.1189798

**Published:** 2016-07-18

**Authors:** Yasuhiko Iwasaki, Jun-ichi Kondo, Akinori Kuzuya, Rui Moriyama

**Affiliations:** ^a^ Department of Chemistry and Materials Engineering; Faculty of Chemistry, Materials and Bioengineering, Kansai University, Suita-shi, Osaka, Japan

**Keywords:** Biomarker, nanogel, phospholipid polymer, oligonucleotide aptamer, 30 Bio-inspired and biomedical materials, 101 Self-assembly/Self-organized materials, 208 Sensors and actuators, 211 Scaffold/Tissue engineering/Drug delivery

## Abstract

Specific detection of protein biomarkers plays an important role in diagnostics and therapeutics. We have fabricated polymeric nanogels, which can specifically interact with the cancer biomarker thrombin to serve as a model. Two types of 2-methacryloyloxyethyl phosphorylcholine (MPC) copolymers bearing a thrombin-binding oligonucleotide aptamer and its complementary chain were independently synthesized by redox-initiated radical polymerization. These MPC polymers associate in a complimentary fashion due to double strand formation of the oligonucleotides in aqueous media, leading to the spontaneous formation of spherical nanogels. Nanogel formation was confirmed by dynamic light scattering (DLS) and transmittance microscopy. The average size of nanogel particles was 124 ± 2 nm and the nanogels were mono-dispersed (polydispersity index 0.21). Functional intercalators could be stably incorporated into nanogels through the physical interaction between the intercalators and the oligonucleotides. The ethidium bromide (EtBr)-incorporating nanogels were used as detectors for thrombin. The fluorescence intensity of solutions containing the EtBr-incorporating nanogels was decreased with an increase in the concentration of thrombin. The transformation of quadruplex–thrombin structure from complementary double-stranded structures resulted in the decrease in fluorescence intensity. In contrast, the intensity did not change when the nanogels were incubated with albumin. Thrombin is only one such model used to demonstrate this technique; oligonucleotide aptamers can be freely designed to interact with versatile bio-substances. Therefore, aptamer-crosslinked nanogels can be appropriate nanomaterials for disease diagnosis and therapy.

## Graphical abstract

**Figure:**
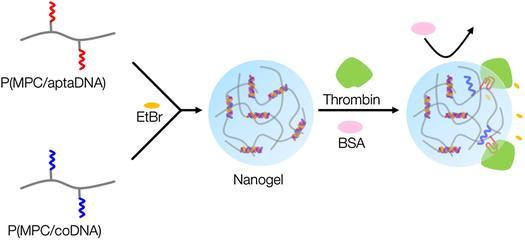

## 1. Introduction

Biomarkers are defined as informative chemicals that enable doctors and researchers to understand a disease state through minimally invasive and simple screening tests.[1] A plethora of biomarkers for cancers have been identified, such as proteins,[2] lipids,[3] carbohydrates,[4] nucleic acids,[5] and cells.[6] Some proteins are related to the type and stage of cancer, and biomarker-based early cancer detection has the ability to decrease deaths from cancer.[7] Recently, multiple technologies for cancer diagnosis using microfluidic devices and nanoparticles have been developed.[8−10] These devices are expected to make progress in the early and reliable detection of cancer. However, several limitations were encountered with these diagnostic systems because biomarkers exist in complex physiological fluids at very low concentrations, which can be sometimes difficult to detect. Furthermore, detection of biomarkers is typically expensive, complex, and frequently requires a time-consuming labeling step, which may induce de-activation of the targeted biomolecule. Therefore, the new development of materials for label-free detection of biomarkers is strongly required.

A major requirement for such biomaterials is the suppression of non-specific bio-fouling to reduce background noise. Several polymers that have anti-fouling properties have been explored. Zwitterionic 2-methacryloyloxyethyl phosphorylcholine (MPC) polymer is one of the most well-studied anti-fouling polymers.[11] MPC is a methacrylic monomer, and thereby a versatile functional co-monomer can be polymerized with MPC. Phosphorylcholine (PC) groups of MPC are highly hydrated with no net charge.[12] Therefore, attractive forces between biomolecules and PC groups are not experienced, leading to a reduction in non-specific bio-fouling on MPC polymers.[13] This property allows the achievement of highly sensitive and selective detection of biomarkers. Upon addition of ligands to MPC polymers, specific molecular interactions between the chemical surface and either proteins or cells can be regulated.[14,15] Several ligands, such as nucleic acids,[16] carbohydrates,[17] peptides [18] and proteins [19] have been immobilized on MPC polymer surfaces. Although these functional MPC polymers have been subsequently applied to diagnostic and drug delivery systems, the application of MPC polymers relative to cancer diagnosis is still not sufficiently studied.

We therefore have designed MPC polymer nanogels for label-free detection of a biomarker in the current study. Nanogels have been recently implicated as drug delivery system (DDS) carriers.[20,21] They have many advantages such as a large surface area, non-cytotoxicity, dispersion in aqueous media, drug loading capacity, sustained drug release, and cellular permeability. Several of these nanogel properties are also beneficial for diagnostic applications. To obtain biomarker-responsive MPC polymer nanogels, the oligonucleotide aptamer was chosen to form a crosslinked structure due to the robust nature of aptamer binding to specific target molecules, which range from small molecules to macromolecules, such as proteins.[22] In addition, oligonucleotide aptamers are versatile and cost-effective.

Thrombin was chosen as the model biomarker in the current study as it is a very important serine protease in the coagulation cascade, which catalyzes the conversion of fibrinogen to fibrin, serving as the thrombus formation.[23] Thrombin generation level is related to solid cancer, and cancer patients are at high risk of venous thromboembolism.[24] Quantification of thrombin is therefore a positive prediction of venous thromboembolism in cancer patients. For label-free detection of thrombin, a thrombin-binding oligonucleotide aptamer (TBA) has shown to provide a robust sensing system.[25,26] TBA consisting of 15-mer DNA (Table 1) was initially discovered by Bock et al. [27]. TBA in complex with thrombin folds as a chair-like quadruplex with two stacked G-quartets, the TGT loop on one side and the two TT loops on the other side.[28]

**Table 1.** Sequences of methacrylic oligonucleotides.

Name	Sequences
aptaDNA	5′- acrydite- TTT TTT TGC TCC**GGT TGG TGT GGT TGG**-3′
coDNA	5′- acrydite-TTT TTT TTT TTT CCA ACC GGA GC-3′

Bold letters: the binding sequence for thrombin, Underlined: complementary DNA sequences.

**Figure 1.**
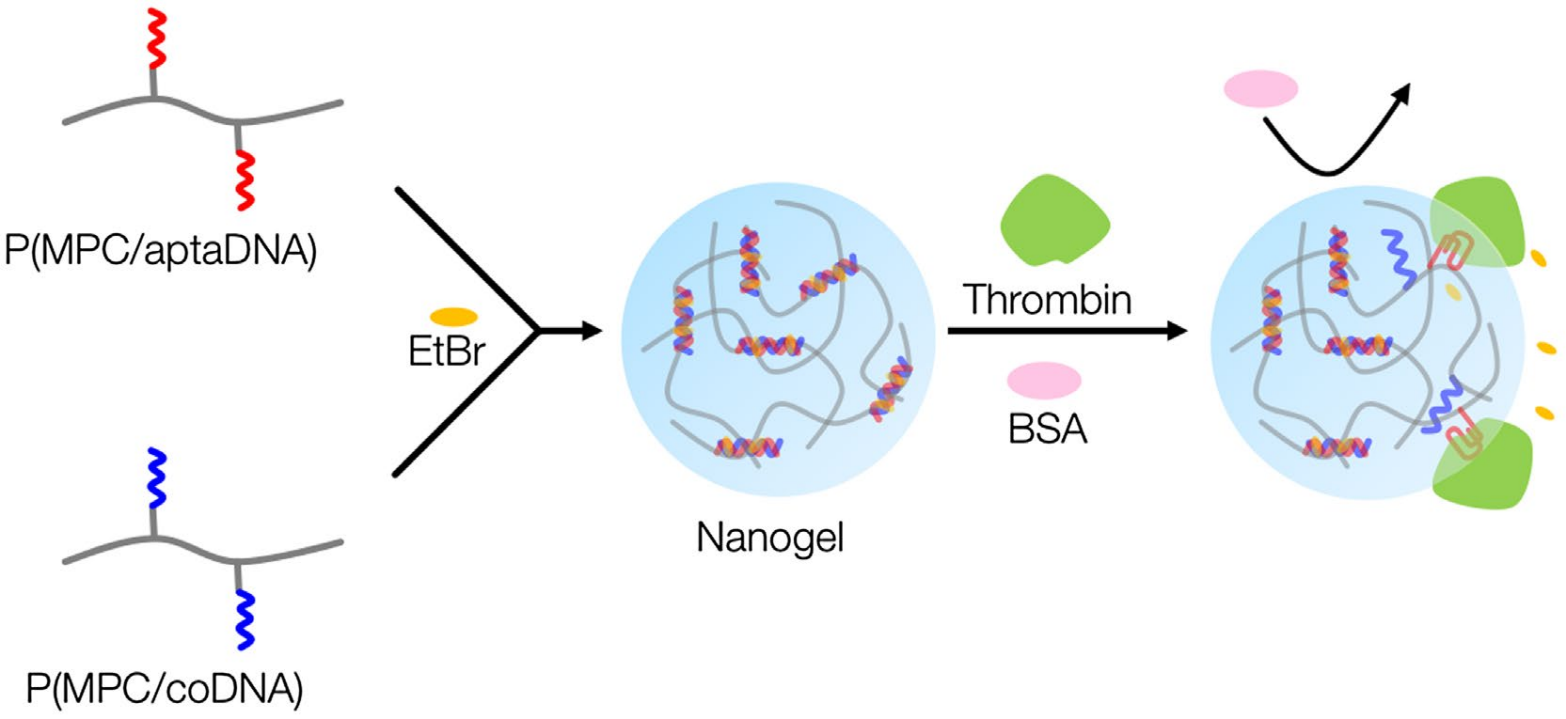
Schematic representation of thrombin detection by MPC polymer nanogels.

Figure 1 shows a schematic representation of thrombin detection by MPC polymer nanogels. MPC polymer nanogels were spontaneously formed by mixing solutions of MPC copolymers bearing TBA and its complementary oligonucleotide chains. The oligonucleotide crosslinked MPC polymer nanogels could selectively monitor thrombin levels without any labeling technique and thus will be valuable tools for cancer therapeutics.

## 2. Materials and methods

### 2.1. Chemicals

2-Methacryloyloxyethyl phosphorylcholine (MPC) was kindly provided by NOF Co., Ltd (Tokyo, Japan) and used without further purification. Methacrylic phosphoramidite was synthesized via the reaction of 6-hydroxyhexyl methacrylamide and 2-cyanoethyl-*N*,*N*,*Nʹ*,*Nʹ*-tetraisopropylphosphoramidite.[29] Other commercially available reagents and solvents were of extra-pure grade and used without further purification.

### 2.2. Synthesis of acrydite-modified oligonucleotides

The oligonucleotides were synthesized on the ABI 394 DNA/RNA Synthesizer (Applied Biosystems, Foster City, CA, USA). Reagents for automated DNA synthesis were purchased from Glen Research Co. (Sterling, VA, USA). For the synthesis of acrydite-modified oligonucleotides, termed aptaDNA and coDNA, capping of methacrylic phosphoramidite to the 5ʹ end of oligonucleotides was also performed on the above-mentioned machine. The acrydite-modified oligonucleotides were cleaved from the support and de-protected by 28% ammonia hydroxide at 60 °C for 24 h. Sequences of aptaDNA and coDNA are shown in Table 1.

Acrydite-modified oligonucleotides were purified by dialysis against phosphate buffered saline (PBS) and water for 6 h each. All modified oligonucleotides were characterized by matrix-assisted laser desorption/ionization time-of-flight mass-spectrometry (MALDI-TOF MS, microflex LRF system, Bruker Daltonics, Bremen, Germany) in the positive ion mode. Concentration of the stock solution of each oligonucleotide was determined with UV absorption of DNA at 260 nm.

### 2.3. Synthesis of MPC copolymers bearing oligonucleotides

**Figure 2.**
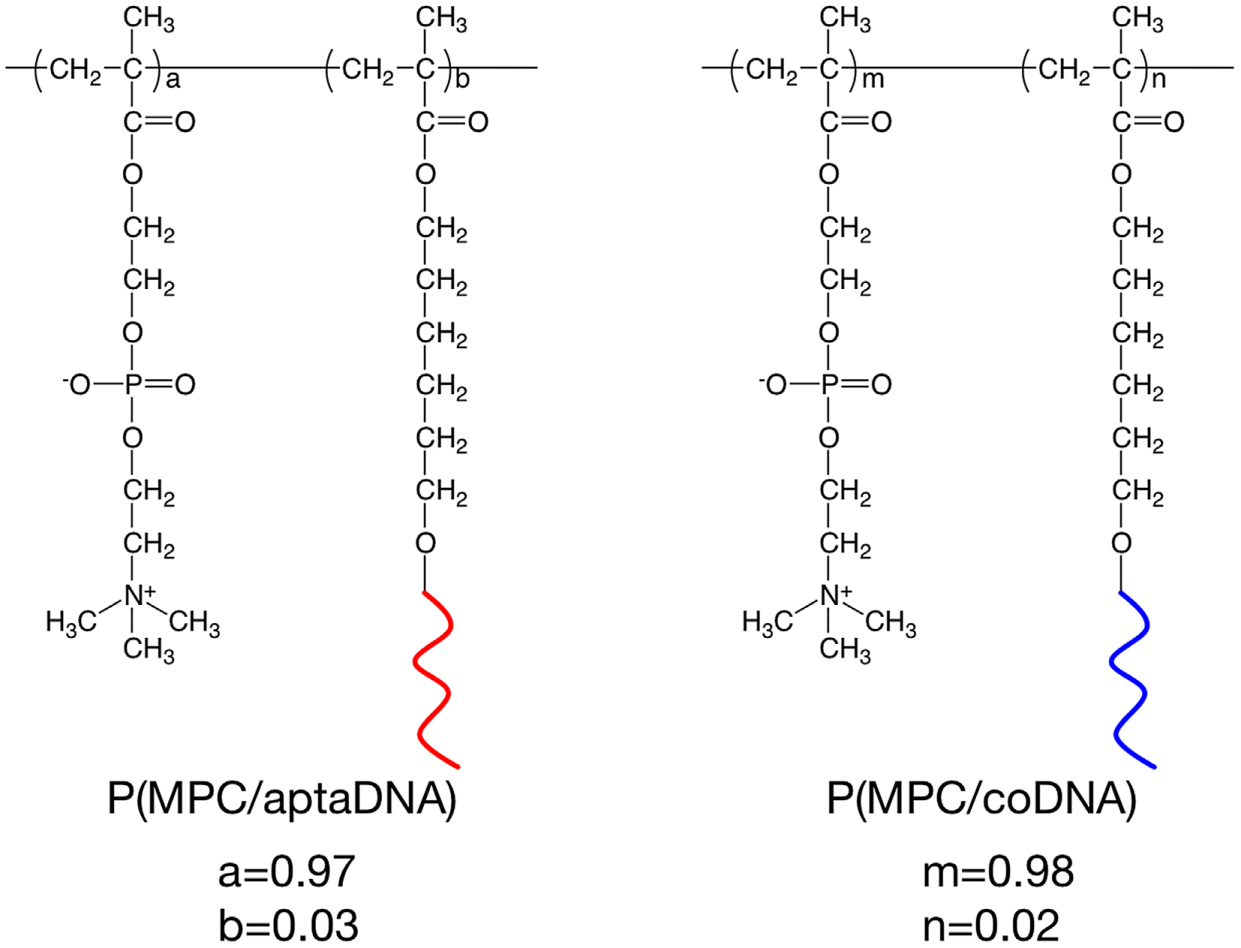
Chemical structures of P(MPC/aptaDNA) and P(MPC/coDNA).

MPC copolymers bearing oligonucleotides P(MPC/aptaDNA) and P(MPC/coDNA), as shown in Figure 2, were synthesized by redox-initiated radical polymerization. MPC (100 mM) and acrydite-modified oligonucleotide (5 mM) were dissolved in PBS. Polymerization was performed using 6 mM ammonium peroxodisulfate (APS) and 60 mM *N*,*N*,*N*ʹ,*N*ʹ-tetramethylethylenediamine (TMEDA). The total volume of the polymerization solution was adjusted to 50 μl. The polymer was purified by gel permeation chromatography (GPC) using a JASCO GPC system, Tokyo, Japan equipped with a refractive index detector and size-exclusion columns, Shodex, SB-803 HQ, and SB-806 M, with a poly(ethylene glycol) (PEG, Tosoh standard sample) standard in distilled water containing 50 mM PBS and 300 mM NaCl. The mole fraction of acrydite-modified oligonucleotides in copolymers was determined by a SYBR® Gold staining assay. P(MPC/aptaDNA) and P(MPC/coDNA) were dissolved in 1 × SYBR® Gold (200 μl) to adjust the concentration to 2 μg ml^–1^ and 4 μg ml^–1^, respectively. The polymer solutions were stored for 1 h. The fluorescence intensity of the solution was measured by a fluorescence spectrophotometer (FP-8300, JASCO). The quantification of acrydite-modified oligonucleotides was calculated from a calibration curve, drawn using the aptaDNA.

Binding of thrombin to P(MPC/aptaDNA) was determined by agarose gel electrophoresis. The P(MPC/aptaDNA) was dissolved into 10 mM PBS containing 10 mM KCl and 150 mM NaCl, and the polymer concentration was adjusted to 500 nM. Thrombin was added to the polymer solution ([thrombin]/[polymer] = 10/1) and the mixed solution was stored for 4 h at 37 °C. The electrophoresis of P(MPC/aptaDNA) and its complex with thrombin was performed at 150 V. Then, the agarose gel was stained with SYBR® Gold and observed by ImageQuant LAS4000 (GE Healthcare Japan, Tokyo, Japan).

### 2.4. Preparation and characterization of nanogels

P(MPC/aptaDNA) or P(MPC/coDNA) was dissolved in PBS at a DNA concentration of 5 μM. The two polymer solutions were mixed together and shaken with a vortex mixer for 20 min at room temperature.

The morphology and size of the nanogels were analyzed by cryo-transmission electron microscopy (TEM; JEM-1400, JEOL, Tokyo, Japan) at 100 kV. Cryo-TEM samples were prepared with ice embedding, which is a type of rapid freeze fixation technique, with Leica EM CPC (Leica Microsystems, Vienna, Austria). A micropipette was used to load 5 μl solution onto a TEM copper grid, which was blotted with filter paper for approximately 2 s, resulting in the formation of thin films suspended on the mesh holes. The samples were quickly plunged into a reservoir of liquid ethane (cooled by liquid nitrogen) at −174 °C. The fixed samples were then stored in liquid nitrogen until they were transferred to a cryogenic sample holder (914 High Liquid Nitrogen Cryo Transfer Tomography Holder, Gatan, Pleasanton, CA, USA) and examined with a JEOL JEM-1400 TEM (100 kV) using minimum dose system (MDS) at approximately −174 °C.

Dynamic light scattering (DLS; ZETASIZER NANO-ZS, Malvern Instruments Ltd, Worcestershire, UK) was also used to analyze the average diameter of particles and the polydispersity index (PDI).

### 2.5. Specific detection of thrombin

Nanogels were prepared as mentioned before. The nanogel solution (10 μl) was mixed with 10 μM ethidium bromide (EtBr; 10 μl), and then the solution was diluted 50-fold with 10 mM PBS containing 10 mM NaCl and 10 mM KCl. After storing the solutions for 60 min at 37 °C, 100 μM thrombin or BSA was added to the original solution to adjust to final protein concentrations of 50, 100, 250, 500, and 1000 nM, respectively. The fluorescence intensity (λ_ex=_260/λ_em=_590 nm) of sample solutions was monitored with a fluorescence spectrophotometer.

## 3. Results and discussion

### 3.1. Synthesis of MPC copolymers bearing oligonucleotides

Acrydite-modified oligonucleotides were established by Boles et al. [30]. Oligonucleotides were covalently immobilized in polyacrylamide hydrogels and specific genes were separated by electrophoresis. After this, several molecular responsive hydrogels have been proposed.[29−34]

In the current study, the acrydite-modified aptaDNA was synthesized by following the sequence as reported by Bock et al. [27]. The synthesis of acrydite-modified oligonucleotides was confirmed by MALDI-TOF MS and the results are shown in supporting Figure S1. The acrydite-modified oligonucleotides were purified by dialysis, yet impurities still remained. However, the methacryloyl group is only introduced to the completed sequence due to unreacted 5ʹ hydroxyl groups, which are protected by acetyl groups using acetic anhydride. Therefore, only the correct chains are used following radical polymerization.

**Figure 3.**
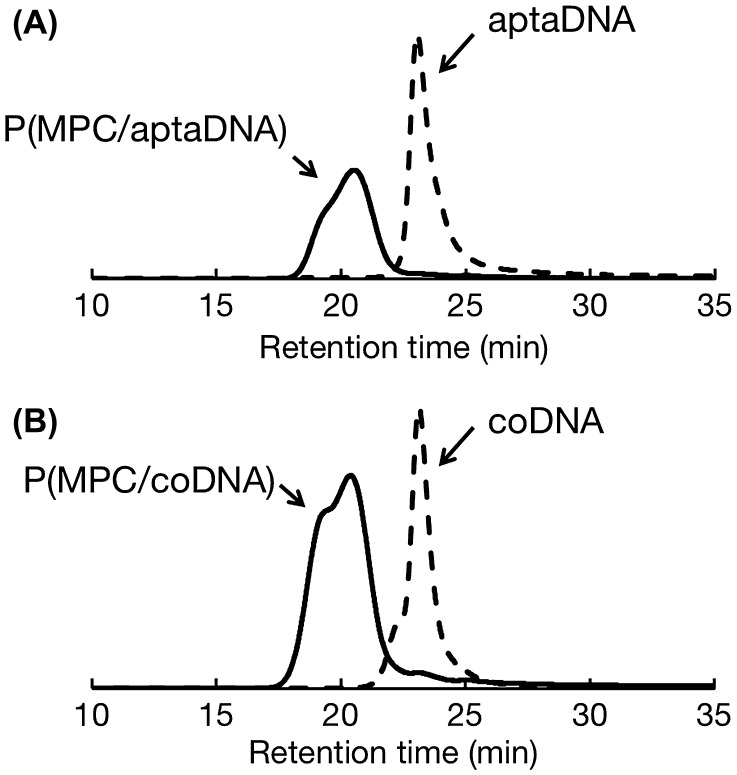
GPC curves of oligonucleotide and MPC copolymers. (A) aptaDNA and P(MPC/aptaDNA); (B) coDNA and P(MPC/coDNA)

MPC and acrydite-modified oligonucleotides were copolymerized by redox-initiated radical polymerization in aqueous media. The chemical structures of P(MPC/aptaDNA) and P(MPC/coDNA) are shown in Figure 2. The molar ratio of MPC to acrydite-modified oligonucleotide in the feed was adjusted at 20/1. In contrast, the molar ratios of MPC to acrydite-modified oligonucleotide were 38/1 (P(MPC/aptaDNA)) and 45/1 (P(MPC/coDNA)). The conversions of P(MPC/aptaDNA) and P(MPC/coDNA) were 32.1% and 34.0%, respectively. Figure 3 shows GPC curves of oligonucleotides and MPC copolymers. The retention times of MPC copolymers were shorter than those of acrydite-modified oligonucleotides. This result indicated that molecular weight increases with acrydite-modified oligonucleotides by polymerization with MPC. The number-averaged molecular weight (*M*_n_) obtained by GPC from MPC polymers was approximately 3 × 10^4^. The density of oligonucleotide chains can be estimated at 1–2 chains per one polymer molecule from the apparent molecular weight. These polymers were stored in a lyophilized state at −30 °C until use.

**Figure 4.**
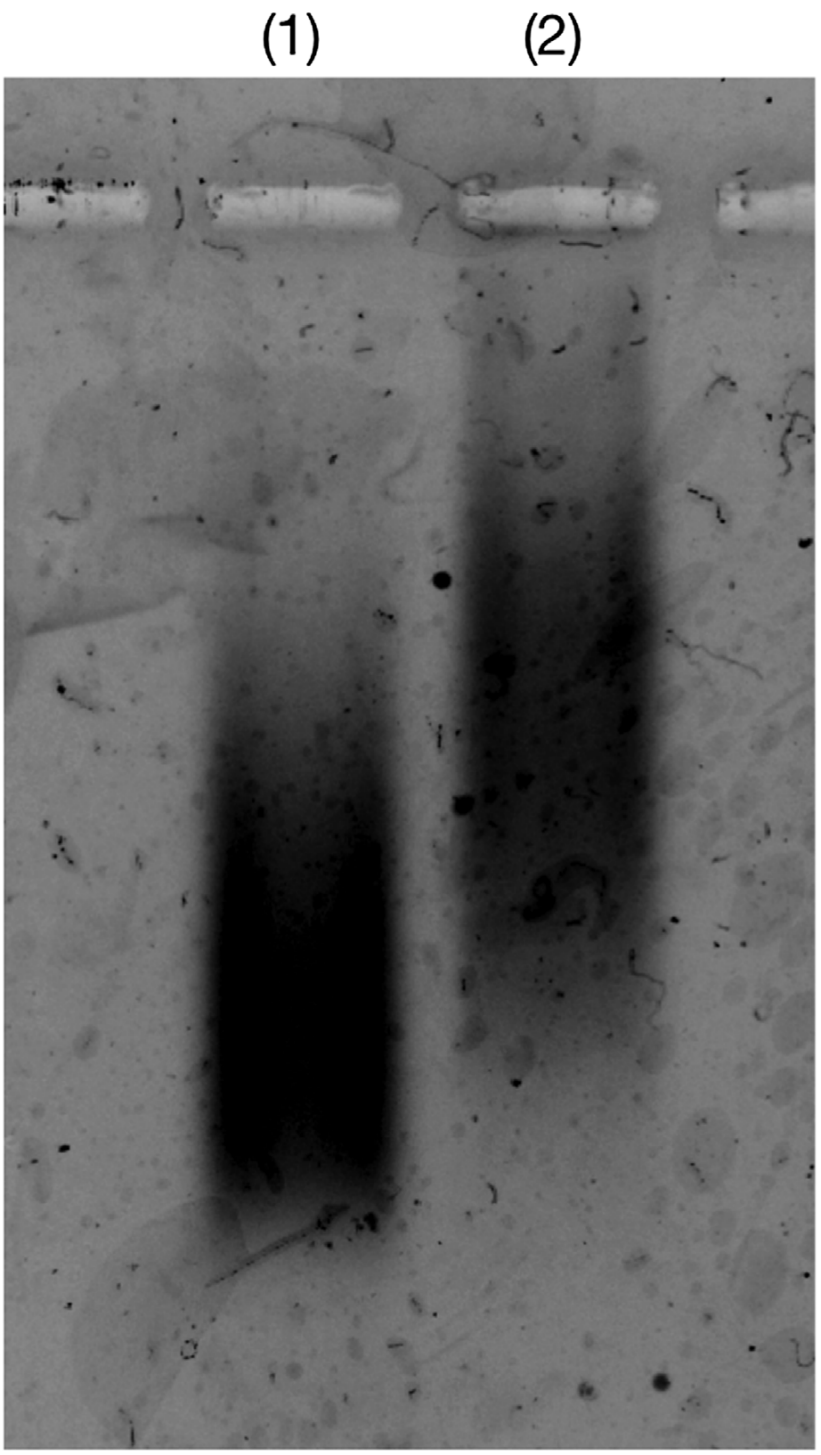
Gel electrophoretic analysis of (1) P(MPC/aptaDNA) and (2) P(MPC/aptaDNA) in contact with thrombin. [P(MPC/aptaDNA)]/[thrombin]=1/10. The gel was stained with SYBR® Gold.

The binding of P(MPC/aptaDNA) and thrombin was confirmed by agarose gel electrophoresis. The molecular weight increment of P(MPC/aptaDNA) after contact with thrombin was observed in Figure 4. This result indicates that copolymerization with MPC does not show an adverse effect on the thrombin binding ability of TBA.

### 3.2. Preparation and characterization of nanogels

**Figure 5.**
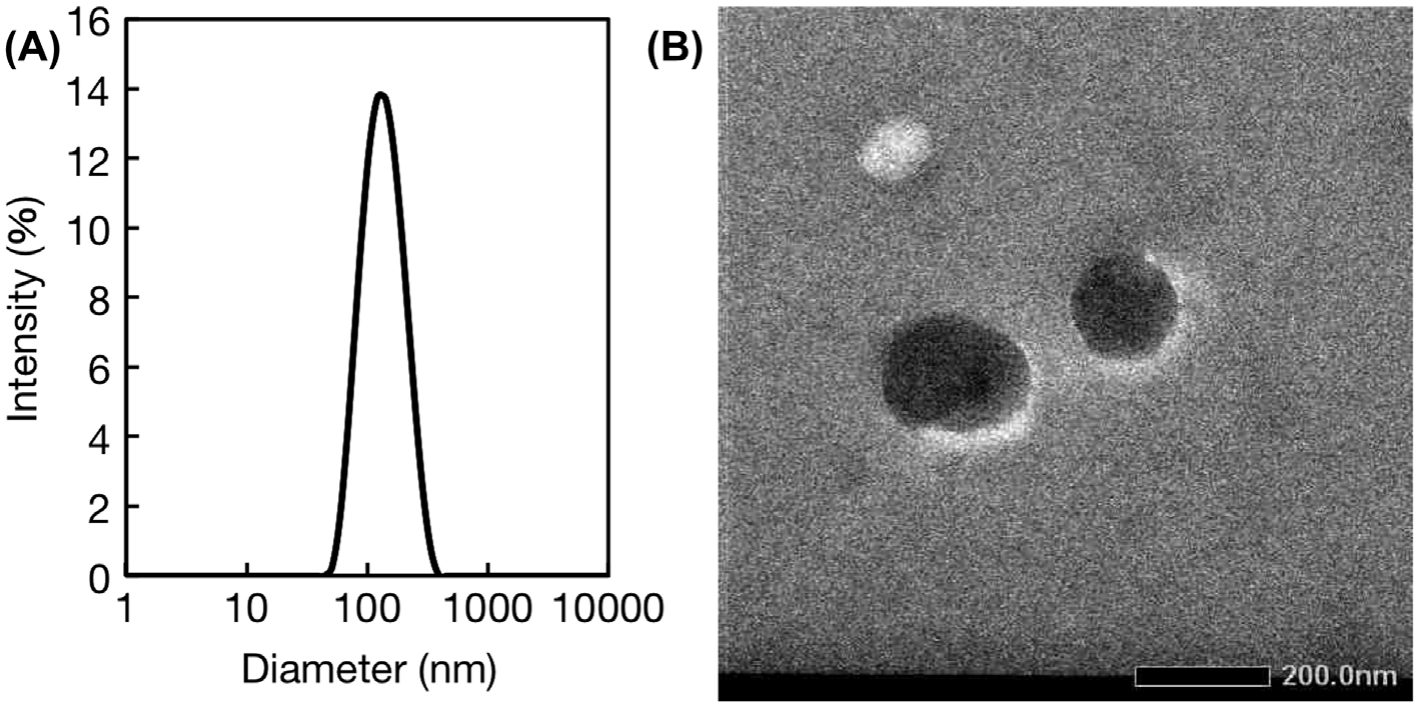
Characterization of nanogels. (A) Size distribution determined by DLS analysis; (B) TEM image.

Nanogels with a physical crosslinking structure of double-stranded oligonucleotides were prepared through simple mixing of 5 μM polymer solutions in an aqueous medium. Figure 5(A) shows a size distribution diagram of P(MPC/aptaDNA) and P(MPC/coDNA) nanogels generated in PBS. The measured hydrodynamic diameters for P(MPC/aptaDNA) and P(MPC/coDNA) were 19.6 and 30.2 nm, respectively (see supporting Figure S2). In contrast, the diameter of unaltered nanogels was 123.6 nm – a much larger diameter owing to their monomeric nature. This result coincides with the TEM analysis as shown in Figure 5(B). The nanogels have a spherical shape. We varied the mole fraction of monomer units in copolymers during the nanogel formation. When the ratio of MPC to acrydite-modified oligonucleotide was 100/1 in the feed, the mono-dispersed nanogel was not formed, as shown in supporting Figure S3. Furthermore, the nanogels were disintegrated after soaking in basic media (pH >14) as shown in supporting Figure S4. This result indicates that nanogel formation occurred through the duplex formation.

Methodologies for nanogel preparation could be divided into physical self-assembly, polymerization, chemical crosslinking of polymers, and templated fabrication.[35] The physical self-assembly of hydrophilic polymers through hydrophobic or electrostatic interactions is the most promising way to modulate nanogels. Akiyoshi et al. [20,21] have synthesized cholesterol-bearing pullulan and applied them as drug carriers and molecular chaperones^[20], [21]^. Tamura et al. [36] prepared PEGylated-polyamine-based polyion complex nanogels for siRNA delivery^[36]^. Physical crosslinked network structures in nanogels show a dynamic nature and are suitable for encapsulation and controlled release of therapeutic agents such as drugs and biomolecules. These nanogels may also provide unique properties in an environmentally responsive manner. However, neither hydrophobic nor electrostatic association is critical for recognition of specific molecules. Oligonucleotide aptamer crosslinked nanogels must exhibit an advantage to recognize the specific target.

### 3.3. Specific detection of thrombin

**Figure 6.**
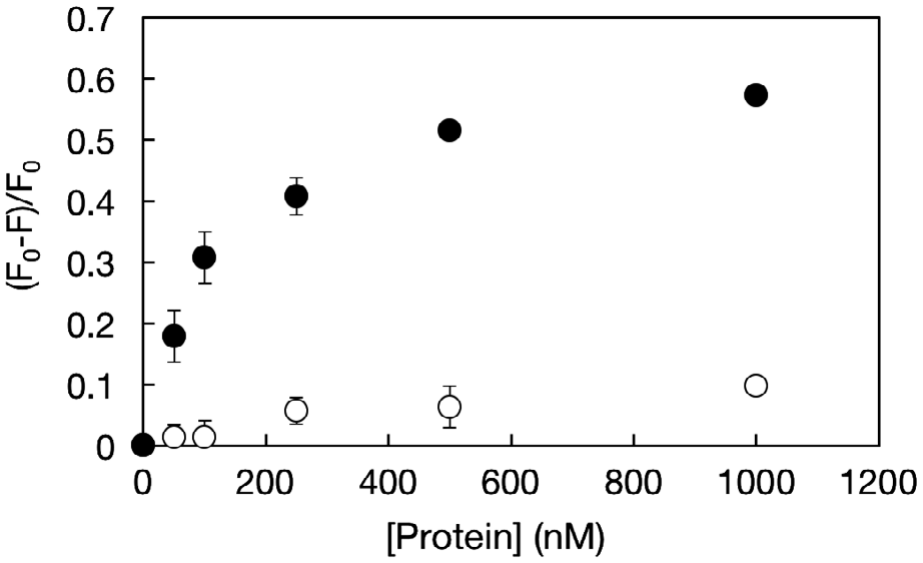
Change in the fluorescence intensity of solution containing EtBr-loaded nanogels after incubation with thrombin (●) or BSA (○). F0 represents for the fluorescence intensity of blank sample, F represents for the fluorescence intensity with proteins.

Multiple systems for detection of thrombin by using TAB have been recently explored.[25,26,37−39] Wang and Wang [38] fabricated aptamer-functionalized hydrogel diffraction gratings on solid supports^[38]^. When thrombin was in contact with the substrate, hydrogel diffraction gratings swelled due to digestion of crosslinked points. The diffraction light allowed correlation among the change in thrombin concentration. Liu et al. [39] also successfully detected thrombin by a sandwich assay carried out on aptamer-immobilized magnetic beads. This system worked well, even in the presence of foreign impurities. Although TBA is reliable for detection of thrombin, a nanogel system has not been well studied yet. Thrombin-induced dissociation of dissociation of the duplex formed between the P(MPC/aptaDNA) and coDNA carrying a fluorescence probe is shown in supporting Figure S5. Bands representing single strand coDNA specifically appeared after incubation with thrombin. Figure 6 shows the change in the fluorescence intensity of solutions containing EtBr-loaded nanogels after incubation with thrombin or BSA. The actual fluorescence spectra are shown in supporting Figure S6. Through the addition of thrombin, the fluorescence intensity considerably decreased and was concentration dependent. In contrast, the fluorescence intensity did not change after incubation with BSA. These results indicate that the nanogels can selectively interact with thrombin. Li et al. [40] clarified the molecular mechanism. When EtBr was free in aqueous solution, the fluorescence intensity of EtBr was quite low because of the efficient quenching of the excited state by transferring an amino proton to the solvent molecule. In contrast, when EtBr was intercalated with dsDNA, it was shielded to some extent and an obvious enhancement in fluorescence intensity was observed.[41] An anti-thrombin aptamer was hybridized with its complementary strand to form double-stranded DNA (dsDNA), after which EtBr was added to produce a relatively high fluorescent signal. According to the formation of complexes containing an aptamer and thrombin, deformation of dsDNA occurred and the fluorescence intensity of EtBr decreased.

## 4. Conclusions

TAB-crosslinked MPC polymer nanogels were successfully obtained through simple mixing of P(MPC/aptaDNA) and P(MPC/coDNA) in aqueous media. The spherical nanogels with average diameters 100–150 nm were spontaneously formed and functional intercalators could be incorporated by integration with dsDNA as a physical crosslinker of the nanogels. Label-free detection of thrombin was performed by EtBr-incorporating TAB-crosslinked MPC polymer nanogels. Oligonucleotide aptamers can be freely designed for interacting with versatile bio-substances.[42] Therefore, aptamer-crosslinked nanogels have potential to be modified for different disease diagnosis and therapeutics.

## Disclosure statement

No potential conflict of interest was reported by the authors.

## Funding

This work was supported by MEXT KAKENHI [grant number 26107719]
